# Effects of temperature on life‐history traits of the newly invasive fall armyworm, *Spodoptera frugiperda* in Southeast China

**DOI:** 10.1002/ece3.7413

**Published:** 2021-03-18

**Authors:** Li‐Li Huang, Fang‐Sen Xue, Chao Chen, Xin Guo, Jian‐Jun Tang, Ling Zhong, Hai‐Min He

**Affiliations:** ^1^ Department of Ecology and Environment YuZhang Normal University Nanchang China; ^2^ Institute of Entomology Jiangxi Agricultural University Nanchang China; ^3^ Department of Entomology and Nematology University of Florida Gainesville FL USA; ^4^ College of Computer and Information Engineering Jiangxi Agricultural University Nanchang China; ^5^ Jiangxi Bureau of Plant Protection and Quarantine Nanchang China

**Keywords:** body weight, development time, fall armyworm, fecundity, growth rate, protogyny

## Abstract

In mid‐May, 2019, the fall armyworm (FAW) *Spodoptera frugiperda* invaded Jiangxi Province, China, and caused extensive damage to corn crops. However, little attention has been given to the life‐history traits of the FAW. In the present study, we systematically investigated the life‐history traits of the newly invasive FAW on corn leaves at 19, 22, 25, 28, and 31°C under a photoperiod of LD 15:9 hr. The FAW thrived on the corn leaves with short developmental periods, high survival rates of larvae and pupae, very high mating success rates, and high fecundity. The pupal developmental stage was significantly longer in males than females at all temperatures, thus resulting in a protogyny phenomenon. The pupal weight was heaviest after a relatively shorter larval development stage at a higher temperature (25°C); thus, the FAW did not follow the temperature–size rule. Females were smaller than males, indicating sexual size dimorphism. A small proportion of females delayed their pre‐oviposition period and began to lay eggs on the 7th to 9th day after adult emergence. There were positive relationships between pupal weight and larval developmental time and between adult weight and fecundity. There was a negative relationship between fecundity and longevity. These findings can help us to predict the population dynamics of the FAW on corn and to develop a suitable and practical management strategy.

## Graphical abstract

Fall armyworm thrived on the corn leaves with short developmental periods, high survival rates, high fecundity, etc., indicating that the species can easily adapt to a new environment wherever it invades.

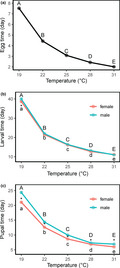

## 1 INTRODUCTION

The fall armyworm (FAW) *Spodoptera frugiperda* (J. E. Smith) is an economically important pest that is native to the tropical and subtropical regions of the Americas, where it is well known as a key pest of corn (Barfield et al., 1978; Garcia et al., 2018; Sparks, 1979). The FAW cannot survive the winter in temperate North America because it cannot enter a diapause. The regions in the United States in which FAW overwinters are southern Florida and southern Texas (Barfield et al., 1978; Johnson, 1987). The moth is a sporadic and long‐distant migratory pest, and an adult can fly over 100 km in a single night (Johnson, 1987). The FAW invaded West and Central Africa in early 2016 (Goergen et al., 2016), Kanataka, India, in early May–June 2018 (Deshmukh et al., 2018), and Jiangcheng County in Yunnan Province in southwestern China in January 2019 (Yang et al., 2019). In mid‐May, the FAW subsequently spread throughout the region of Jiangxi Province, Southeast China (from 24.5°N, 113.6°E to 30.08°N, 118.5°E), and caused significant crop losses in corn, which was more severe than the damage caused by the Asian corn borer *Ostrinia furnacalis*. The FAW larvae feed on young leaf whorls, ears, and tassels. The late‐instar larvae can cut through the base of corn seedlings, thus killing the whole plant. Although the FAW displays a very wide host range, one of its main host plants in Jiangxi Province is field corn (*Zea mays* L.).

Temperature is considered as one of the most important environmental variables that induce phenotypic plasticity in ectotherms and affects various aspects of life‐history traits of insects (Atkinson, 1994). Generally, an increase in temperature, within a favorable range, increases metabolic rate and consequently results in higher growth rate, shorter development time, and smaller adult body size (Sibly & Atkinson, 1994). This phenomenon has been called the “temperature‐size rule.” Atkinson (1994, 1995) estimated that >80% of ectothermic species studied in laboratories exhibited faster growth but smaller adult body size at higher rearing temperatures.

The life‐history traits of an insect, such as its developmental time, body size, fecundity, and longevity, are typically of great importance to its fitness (Nylin & Gotthard, 1998). Whether a pest outbreak occurs is also closely related to its life‐history traits. Thus, the development of practical management strategies for the newly invasive FAW should be based on knowledge of its life‐history traits in the local environment. The life‐history traits of the FAW have been investigated in the Western Hemisphere (Ali et al., 1990; Barfield et al., 1978; Barros et al., 2010; Garcia, 2018; Johnson, 1987), where the FAW shows a relatively short generation time, a high reproductive rate, and good dispersal ability. However, information on the life‐history traits of the newly invasive FAW in the Eastern Hemisphere is still scarce, and only He et al. (2020) investigated the influence of larval diet on the development and reproduction of the newly invasive FAW. It is necessary to investigate how the environmental conditions affect FAW life‐history traits and to evaluate the thermal fitness of FAW in newly invaded regions.

In the present study, we systematically investigated the development, survival rate, mating success, and fecundity of FAW on corn leaves at five different temperatures (19, 22, 25, 28, and 31°C) to assess the effects of temperature on the life‐history traits of FAW.

## 2 MATERIALS AND METHODS

### 2.1 Insect material and culture

More than 100 full‐grown larvae of *S. frugiperda* were collected from different corn fields in Yongxiu County (29°04′N, 115°82′E), Jiangxi Province, China, in early July 2019. The larvae collected were individually reared in Petri dishes (height 2.0 cm; diameter 9.0 cm) containing moistened filter paper and a fresh corn leaf (*Z. mays*) until adult eclosion under natural conditions. The Petri dishes were checked daily and supplied with new fresh leaves when needed (the same below). On the day of adult emergence, the adults were sexed and pairs of males and females were transferred into sealed plastic bags (20 × 30 cm) filled with air for mating and oviposition. Cotton balls moistened in sugar solution (10% honey mixed in 90% water by volume) were placed in the sealed plastic bags to nourish the adults (the same below). The egg masses were collected daily in Petri dishes and were used to conduct the experiments. The eggs were observed daily until hatching.

### 2.2 Experimental design and measurement methods

After hatching, larvae were transferred to 20 transparent plastic boxes (16 × 11 × 5.5 cm) and reared on corn leaves collected at the jointing‐booting stage with 35–70 larvae in a box. The boxes were randomly divided into five groups (four boxes for each group), which were placed in five illuminated incubators (LRH‐250‐GS, Guangdong Medical Appliances Plant) with constant temperatures of 19, 22, 25, 28, and 30 ± 1°C and a photoperiod of LD 15:9 (15 hr light:9 hr dark). After development to the second instar, the larvae were individually transferred from boxes to Petri dishes (height 2.0 cm; diameter 9.0 cm) and maintained their respective temperatures. Each larva was reared in a Petri dish containing moistened filter paper and a fresh corn leaf until adult eclosion. Larvae from one box were regarded as a replicate, and there were four replicates for each temperature condition.

For the studies on egg development at different temperatures, eggs collected within 1 hr after oviposition were placed in Petri dishes lined with moist filter paper and maintained in five illuminated incubators. There were 3–4 replications with 70 eggs each for each temperature condition.

The following traits were recorded for all individuals: egg, larva, and pupa developmental times; pupal and adult weight. Larva and pupa survival rates were counted at each temperature. The pupae were weighed on the day following pupation, and adults were weighed after the release of the meconium. We used an electric balance (AUY120, SHIMADZU Corporation). The growth rate was calculated as ln (pupal weight)/larval development time (Gotthard et al., 1994). The weight loss between pupation and adult eclosion was calculated using the formula: proportional weight lost = 1 − (adult weight/pupal weight).

After the adults emerged from the different temperature treatments, each of 25 pairs of moths for each temperature was transferred to sealed plastic bags with cotton balls moistened in sugar solution for mating and oviposition. The adults were maintained in a rearing room at 26 ± 2°C, 70 ± 15% RH, and natural day lengths. The egg masses were collected daily, and the number of eggs was counted. The plastic bags were replaced daily until the adult died. Adult developmental metrics, including the mating success, preoviposition and oviposition periods, fecundity, and adult longevity, were recorded.

Because the survival rate of pupae was relatively low at 19°C and the dead pupae were relatively small, these data were not used to compare the influence of temperature on adult weight.

### 2.3 Statistical analysis

ANOVA was used for analysis of life‐history traits in relationship to temperature and sex. A generalized linear model with binomial error distribution was used to analyze the proportional data (survival and mating success). ANOVA was used to analyze the effect of larval rearing temperature on preoviposition time, oviposition time, and female fecundity. A linear mixed model using the *lme4* package (Bates et al., 2015) was used to test the relationship between larval development time and pupal weight with pupal weight as the response variable and larval time as the explanatory variable and a cohort of insects (larvae hatched from eggs collected in the same day) as the random factor. To test the relationships between adult weight, fecundity, and longevity, we first fitted a linear mixed model for each trait in response to rearing temperature (Figures S1 and S2); for the relationship between adult weight and fecundity, fecundity was treated as the response variable and adult weight as the explanatory variable and cohort of insect as the random factor; for the relationship between adult weight and fecundity, fecundity was treated as the response variable and adult weight as the explanatory variable and a cohort of insects as the random factor; for the relationship between adult fecundity and longevity, longevity was treated as the response variable and adult fecundity as the explanatory variable and a cohort of insects as the random factor; then, the residuals of each model were extracted and used to analyze the general relationship between female adult weight and fecundity as well as female longevity and fecundity. All the analyses were conducted in R software (version 3.4.4) (R Core Team, 2018).

## 3 RESULTS

### 3.1 Developmental time, survival rate, and sex ratio

**FIGURE 1:**
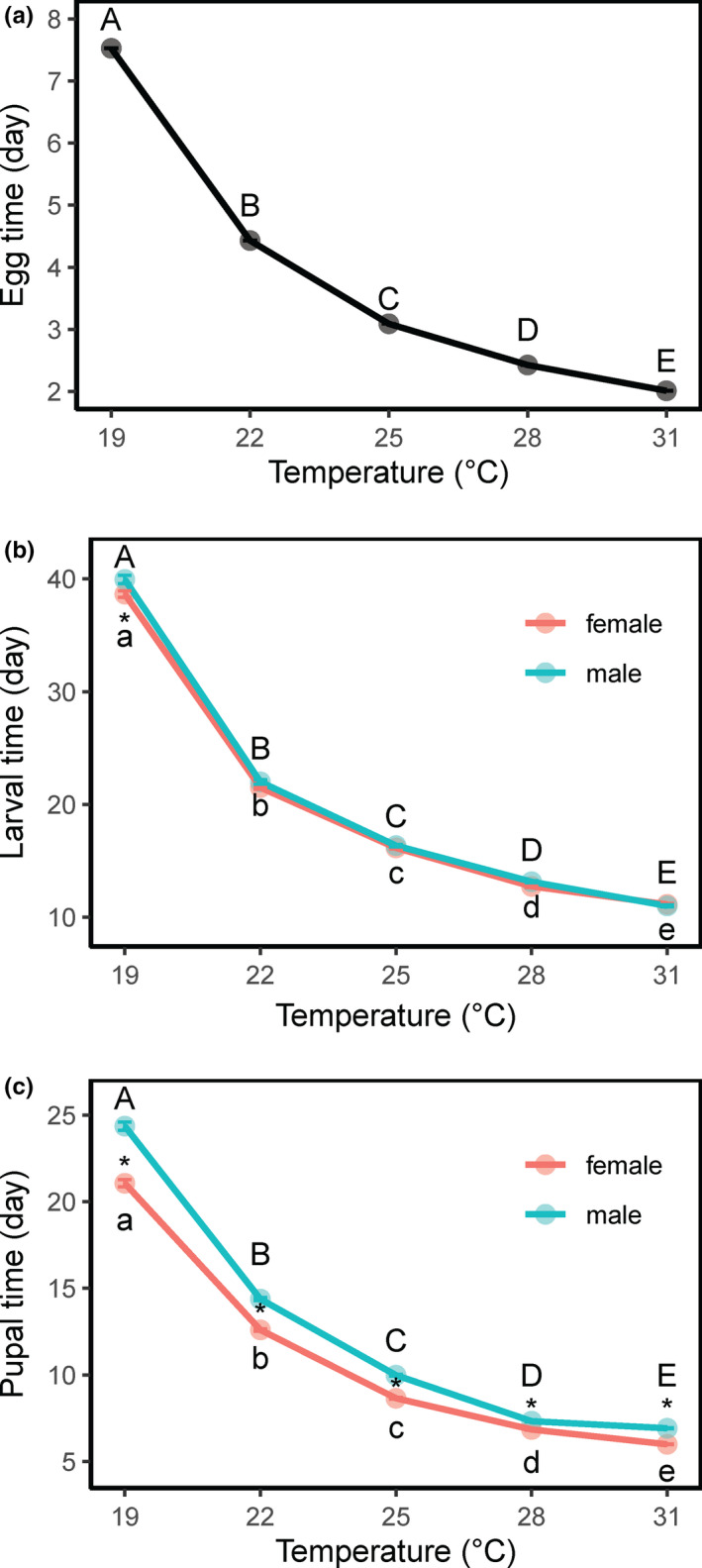
Egg, larval, and pupal development times of the fall armyworm, *Spodoptera frugiperda*, at different temperatures. Error bars indicate *SE*

Temperature significantly influenced the developmental cycle of the insect from egg to adult (Figure 1). The incubation period of eggs decreased significantly from 7.5 days at 19°C to 2.0 days at 31°C (*F*_4,11_ = 182,143, *p* < 0.001) (Figure 1a). The larval development time significantly decreased as the temperature increased (*F*_4,1031_ = 8,481.6, *p* < 0.001) in both females and males; larval development took 39 days for females and 40 days for males at 19°C and 11.1 days for females and 11 days for males at 31°C (Figure 1b, see also Table S1). There were no significant differences in larval developmental time between the sexes at any temperature, except at 19°C (*F*_1.1121_ = 8.36, *p* < 0.05). Pupal development took 21.1 days for females and 24.4 days for males at 19°C and 6 days for females and 6.9 days for males at 31°C (temperature effect: *F*_4,1031_ = 10,994.7, *p* < 0.001) (Figure 1c, see also Table S1). There were significant differences in pupal period between the sexes at all temperatures (sex effect: *F*_4,1031_ = 831.8, *p* < 0.001), with significantly longer time in males than in females, indicating a protogyny phenomenon. This protogyny phenomenon was more pronounced at the low temperature (19°C) than at the high temperature (31°C), with females emerging 3.3 days earlier than males at 19°C and 0.9 days earlier than males at 31°C (see Table S1).

**FIGURE 2:**
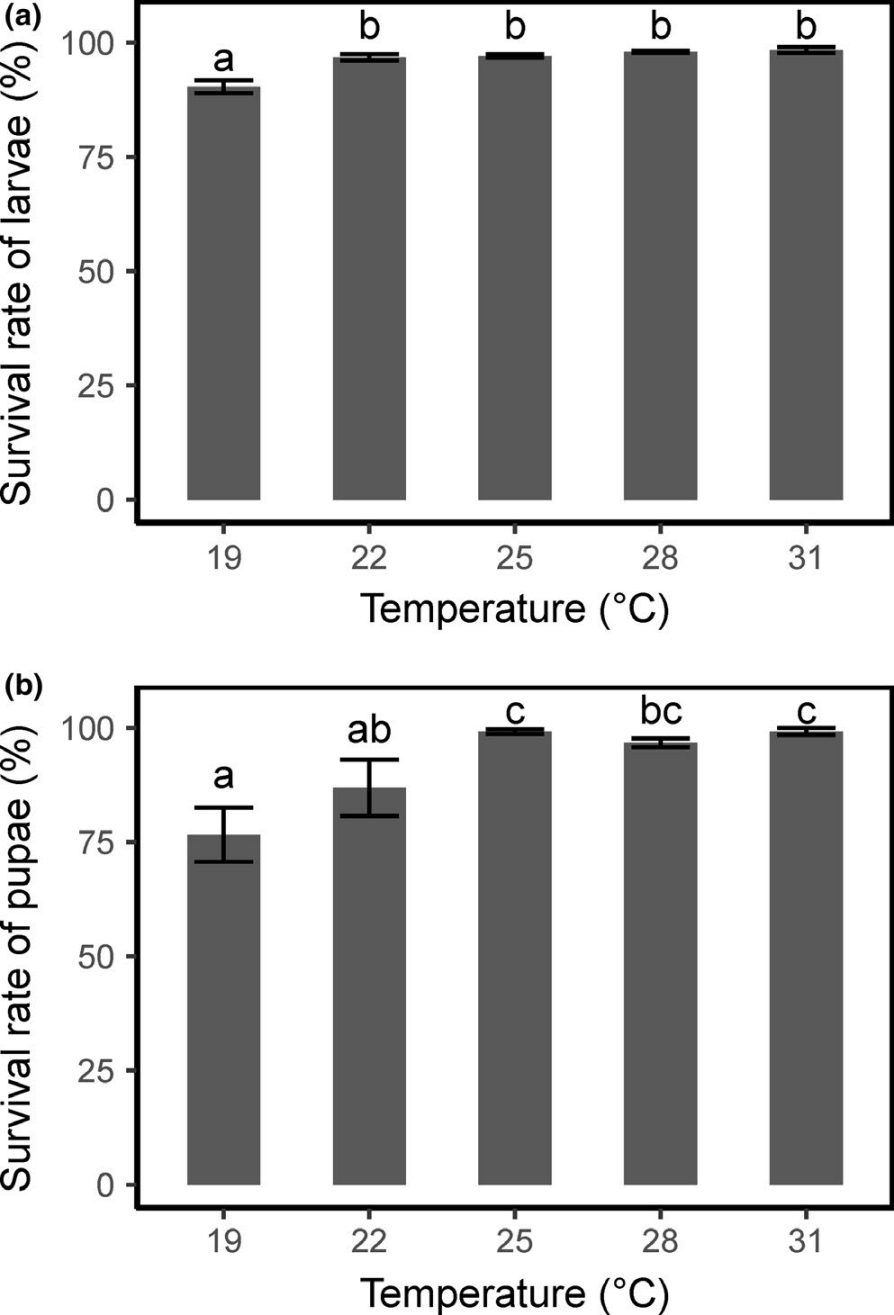
Survival rates of larvae and pupae of the fall armyworm, *Spodoptera frugiperda*, at different temperatures. Error bars indicate *SE*

The survival rate of larvae was 90.4% at 19°C (Figure 2a), which was significantly lower (*χ* ^2^ = 67.6, *df* = 4, *p* < 0.001) than that at other temperatures (96.8%–98.4%). The survival rate of pupae was 76.6% at 19°C, significantly lower (*χ* ^2^ = 21.9, *df* = 4, *p* < 0.001) (Figure 2b) than that at other temperatures (86.9%–99.2%).

**FIGURE 3:**
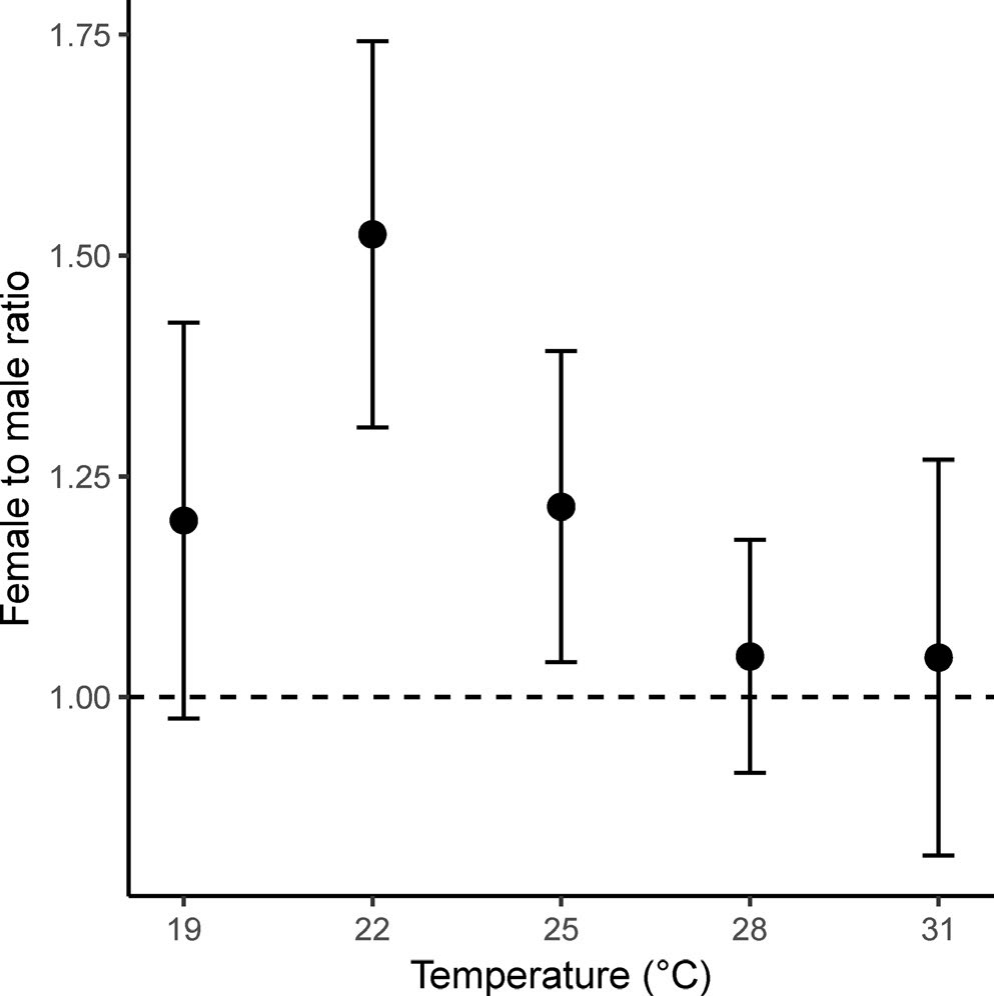
Sex ratio of the fall armyworm, *Spodoptera frugiperda*, at different temperatures. Error bars indicate *SE*

Even though the ratio of females to males was higher at 22°C than at the other temperatures, no significant difference was detected (*χ* ^2^ = 3.4, *df* = 4, *p* > 0.05) (Figure 3). The sex ratios (female:male) were 53:47 at 19°C, 59.4:40.6 at 22°C, 54.1:45.9 at 25°C, 51.1:48.9 at 28°C, and 50.2:49.8 at 31°C.

### 3.2 Growth rate, pupal and adult weight, and weight loss

**FIGURE 4:**
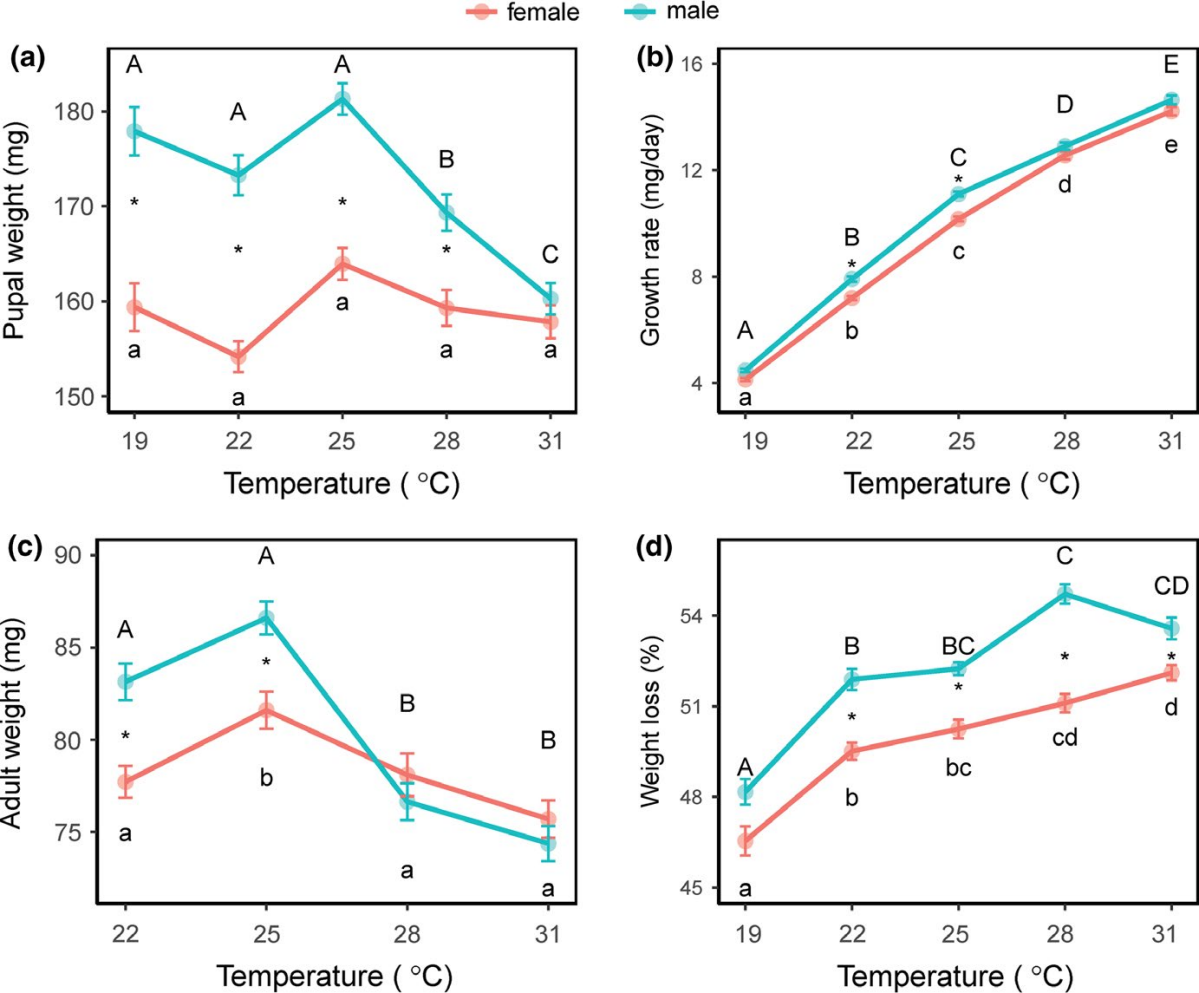
Pupal and adult weight, larval growth rate, and weight loss in the fall armyworm, *Spodoptera frugiperda*, at different temperatures. Error bars indicated *SE*

The growth rate increased significantly as the rearing temperature increased (*F*_4,1031_ = 1,711.3, *p* < 0.001), and the growth rate was higher in males than in females (Figure 4c, see also Table S1). Significant differences in growth rate between the sexes were found at 22 and 25°C (for 22°C: *F*_1, 217_ = 5.0, *p* < 0.001; for 25°C: *F*_1, 248_ = 54.5, *p* < 0.001).

Pupal weight was significantly affected by temperature (*F*_4, 552_ = 4.42, *p* < 0.001). It was highest at 25°C (163.9 mg for females; 181.3 mg for males), followed by those at 19°C (159.4 mg for females; 177.9 for males), 22°C (154.2 mg for females; 173.3 for males), 28°C (159.3 mg for females; 169.3 for males), and 31°C (157.8 mg for females; 160.3 for males) (Figure 4a, see also Table). However, pupal weight did not follow the temperature–size rule (a negative relationship between size and rearing temperature). The pupal weight of females was significantly lower than that of males at all temperatures (for all temperatures, *p* < 0.05) except at 31°C (*p* > 0.05), indicating sexual size dimorphism.

Temperature also significantly influenced adult weight (for females: *F*_4, 552_ = 9.6, *p* < 0.001; for males: *F*_4, 476_ = 45.0, *p* < 0.001) (Figure 4b, see also Table S1). The highest body weight was observed at 25°C (81.6 mg for females; 86.6 mg for males), and the lowest at 31°C (75.7 mg for females; 74.4 mg for males). However, the change in adult weights with temperature did not completely follow the pattern of the change in pupal weights. At 22 and 25°C, females were significantly smaller than males (at 22°C: *F*_1, 217_ = 17.7, *p* < 0.001; at 25°C: *F*_1, 248_ = 14.0, *p* < 0.001). However, females were slightly larger than males at 28 and 31°C although no significant difference was found between the two temperatures.

Weight losses from pupae to adults increased significantly as the rearing temperature increased (temperature effect: *F*_4, 1031_ = 73.0, *p* < 0.001 and sex effect: *F*_1, 1031_ = 125.9, *p* < 0.001) (Figure 4d, see also Table S1). Male pupae lost significantly more weight at metamorphosis than female pupae at 22, 25, 28, and 31°C (temperature × sex effect: *F*_4, 1031_ = 3.6, *p* < 0.05). Thus, female adults were slightly larger than male adults at 28 and 31°C because male pupae lost significantly more body weight than female pupae.

### 3.3 Biology of mated females

Table 1 shows the biology of mated females. The mating success rate was the lowest (27%) at the rearing temperature of 19°C and was significantly lower than that at other rearing temperatures (96%–100%) (*χ* ^2^ = 37.2, *df* = 4, *p* < 0.001).

**TABLE 1:** Biology of mated females of *Spodoptera frugiperda* when newly emerged adults from different temperatures were transferred to a rearing room at 26 ± 2°C and a natural photoperiod for mating and oviposition

Rearing temperature (°C)	Number of mated females	Mating success rate (%)	Preoviposition period (d)	Oviposition period (d)	Longevity (d)	Number of eggs per mated female
19	7	27 ± 7.6 a	5.8 ± 0.8 ab	5.1 ± 0.7 a	12.4 ± 0.9 ab	794.5 ± 64.1 a
22	25	100 ± 0.0 b	4.9 ± 0.3 ab	8.1 ± 0.5 b	14.4 ± 0.8 a	1,555.0 ± 66.6 bc
25	25	100 ± 0.0 b	4.6 ± 0.2 a	7.4 ± 0.3 ab	12.7 ± 0.5 ab	1,671.7 ± 77.4 b
28	24	96 ± 7.0 b	5.8 ± 0.4 b	7.6 ± 0.5 ab	14.6 ± 0.9 a	1,279.0 ± 81.7 c
31	24	96 ± 7.0 b	4.1 ± 0.3 a	6.5 ± 0.5 ab	11.5 ± 0.7 b	1,432.7 ± 81.1 bc

Note

Values (±*SE*) within one row followed by different letters are significantly different at the 0.05 level based on one‐way ANOVA and Tukey's HSD multiple tests.

The preoviposition period was significantly affected by the rearing temperature (*F*_4, 101_ = 5.2, *p* < 0.001), and the shortest preoviposition period (4.1 days) occurred at the rearing temperature of 31°C. It is worth mentioning that a small proportion of females delayed the preoviposition period regardless of the rearing temperature and began to lay eggs on the 7th to 9th day after adult emergence (two out of 24 females at 31 and 28°C, two out of 25 females at 25 and 22°C, one out of seven females at 19°C). For example, one female began to lay eggs on the 7th day after emergence at 31°C and laid 1,164 eggs in her lifetime; one female began to lay eggs on the 9th day after emergence at 28°C and laid 1,159 eggs; one female began to lay eggs on the 8th day after emergence and laid 1,260 eggs at 25°C; one female began to lay eggs on 9th day after emergence at 22°C and laid 1,019 eggs; and one female began to lay eggs on the 8th after emergence at 19°C and laid 710 eggs. The oviposition duration was significantly different among rearing temperatures (*F*_4,101_ = 3.6, *p* < 0.01), with the longest oviposition period (8.1 days) at the rearing temperature of 22°C. There were significant differences in FAW longevity among temperatures (*F*_4,101_ = 3.3, *p* < 0.05), and the shortest longevity was observed at the rearing temperature of 31°C.

Temperature significantly affected female fecundity (*F*_4,101_ = 10.9, *p* < 0.001). Fecundity was highest at the rearing temperature of 25°C and the lowest at the rearing temperature of 19°C, with a mean of 1,671.7 and 794.5 eggs per female, respectively. The highest fecundity for one female was found at the rearing temperature of 28°C, with 2,679 eggs. The highest egg production per day was found at the rearing temperature of 25°C, with 1,151 eggs.

### 3.4 Relationship between two traits

**FIGURE 5:**
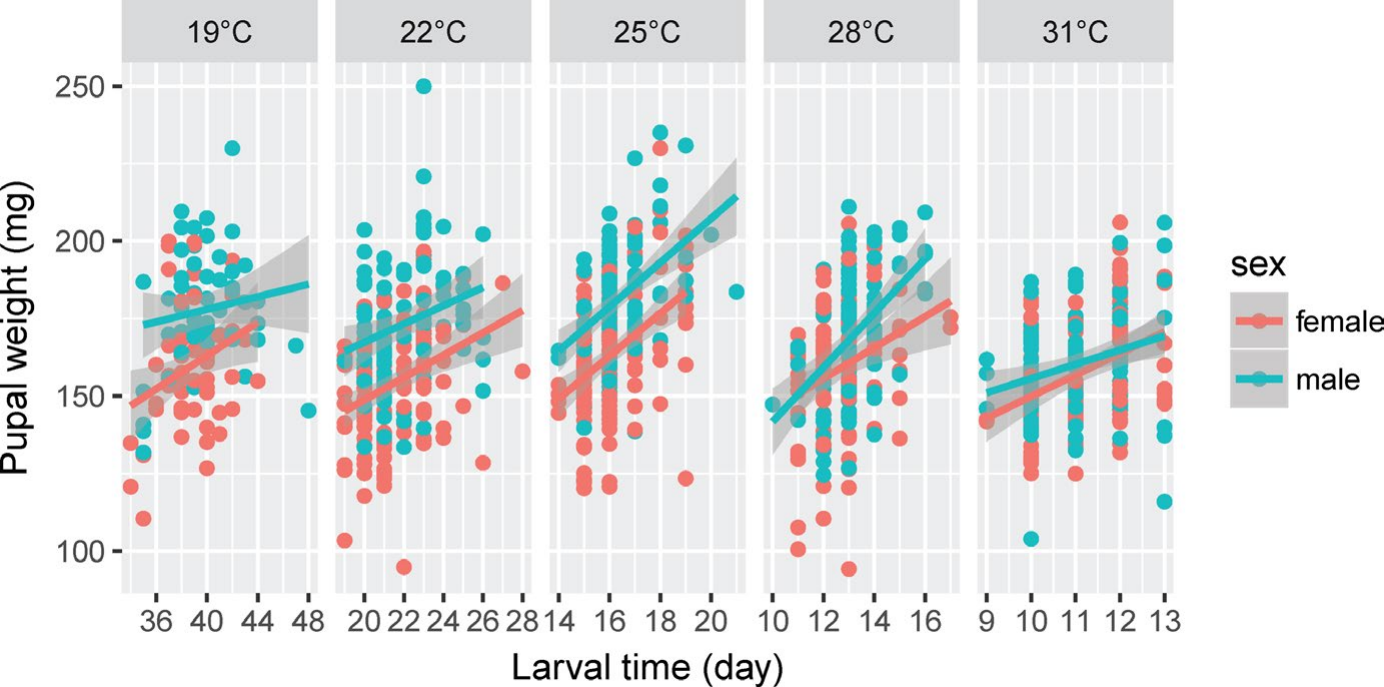
Relationship between pupal size and larval developmental time in the fall armyworm, *Spodoptera frugiperda*, at different temperatures

**FIGURE 6:**
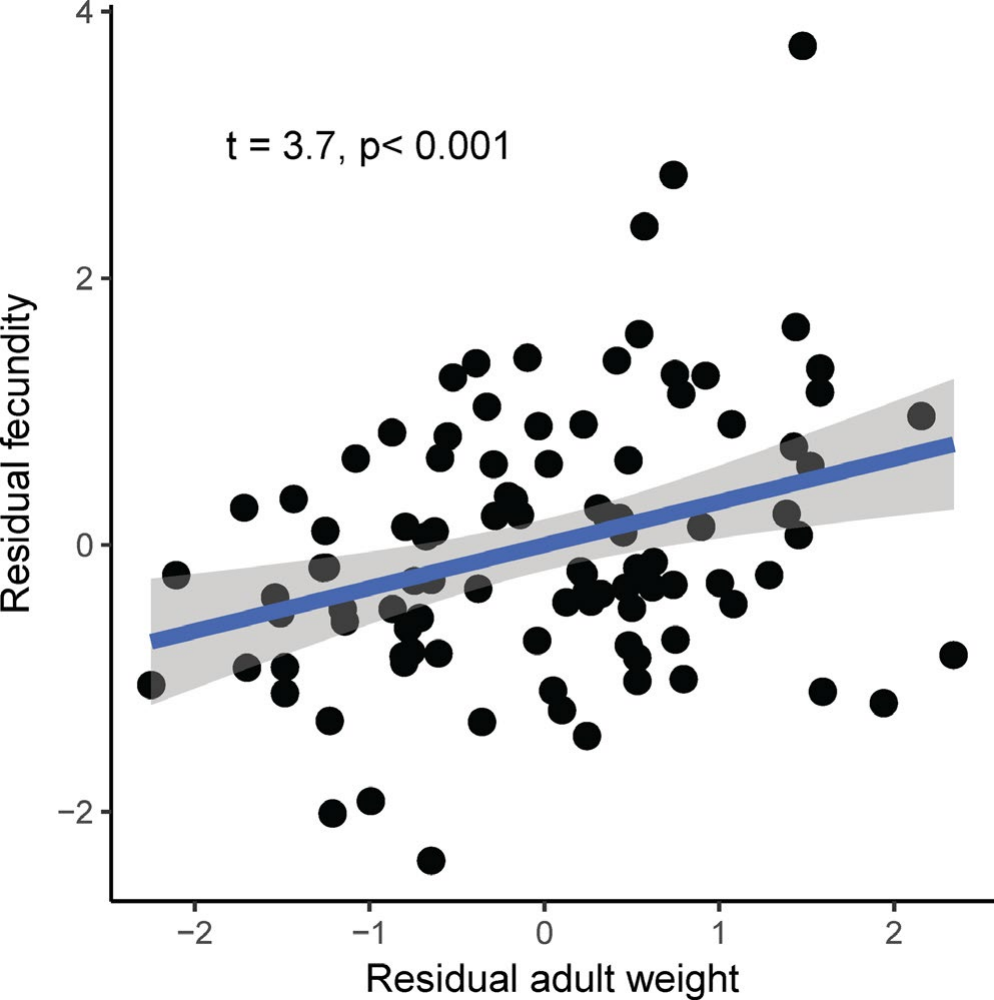
Relationship between female fecundity and adult weight in the fall armyworm, *Spodoptera frugiperda*, when the data from all temperatures were pooled together

**FIGURE 7:**
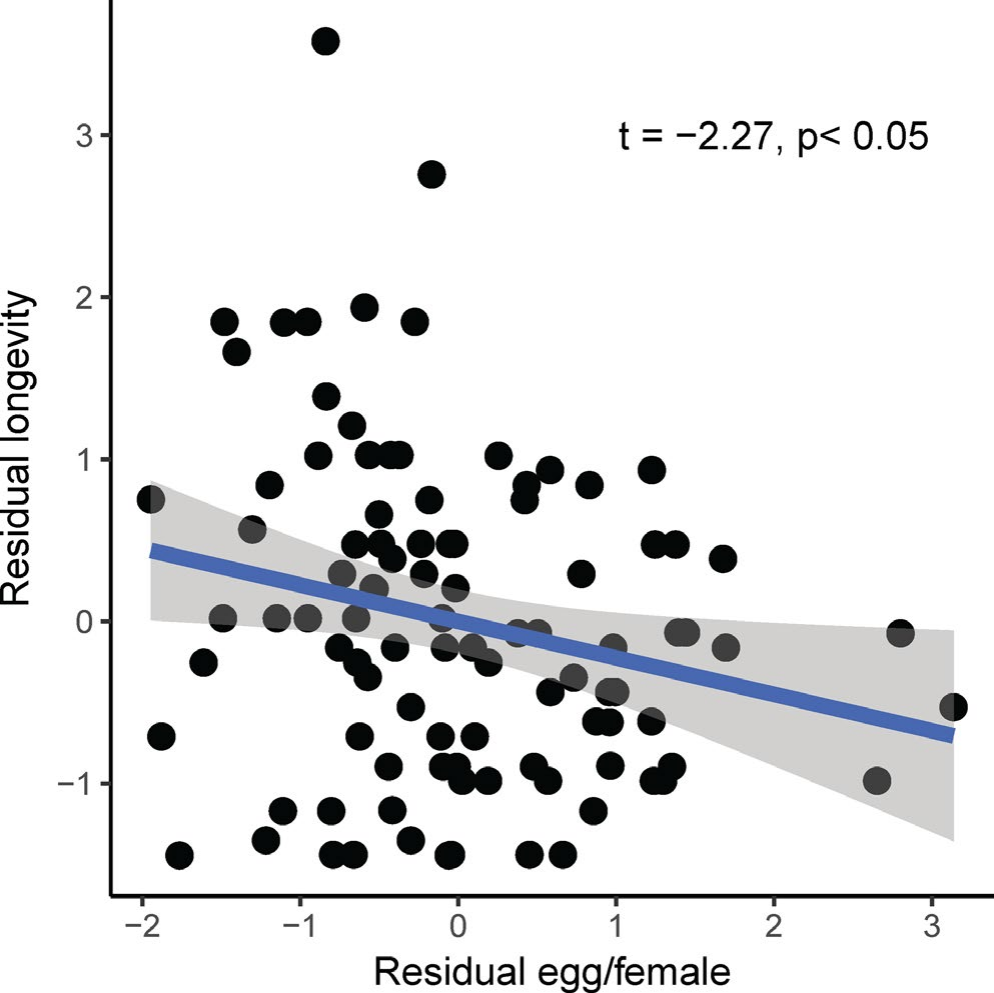
Relationship between female longevity and fecundity in the fall armyworm, *Spodoptera frugiperda*, when the data from all temperatures were pooled together

A significant positive relationship was detected between pupal weight and larval development time at all temperatures, except in males at 19°C (Figure 5, Table 2, *p* < 0.05). There was a significant positive relationship between adult weight and fecundity (Figure 6, *t* = 3.7, *p* < 0.001) and a significant negative relationship between fecundity and longevity (Figure 7, *t* = −2.27, *p* < 0.05).

**TABLE 2:** Relationship between larval time and pupal weight of *Spodoptera frugiperda* was estimated by a linear mixed model, with pupal weight as the response variable, larval time as a fixed factor, and cohort as the random factor

Temperature (°C)	SEX	Estimate	*df*	*t*	*p*
19	Female	2.70 ± 1.11	60.0	2.44	0.0178
	Male	1.01 ± 0.93	53.0	1.10	0.28
22	Female	3.02 ± 0.88	130.0	3.43	0.0008
	Male	2.43 ± 1.10	85.8	2.21	0.03
25	Female	6.85 ± 1.28	129.1	2.44	3.49e‐07
	Male	7.13 ± 1.32	114.0	5.40	3.7e‐07
28	Female	4.96 ± 1.59	115.8	3.11	0.00233
	Male	8.64 ± 1.54	108.4	5.63	1.46e‐07
31	Female	7.01 ± 1.63	108.9	4.30	3.75e‐05
	Male	4.60 ± 1.54	108.0	2.98	0.00353

## 4 DISCUSSION

Successful colonizer species have a high reproductive rate, short generation time, and strong dispersal ability (Dingle, 1972). In the present study, the newly invasive FAW clearly possesses traits that make it a successful colonizer species. Compared with the life‐history traits of the native FAW (also fed on corn leaves) observed by Garcia et al. (2018) (see their data in their Tables 1 and 2), the duration of larval development of the invasive FAW was shorter in the current study at all similar temperatures (11.1 ± 0.1 days at 31°C compared with 14.6 ± 0.1 days at 30°C; 16.3 ± 0.1 days at 25°C compared with 18.3 days at 26°C; 22 ± 0.2 days compared with 27.5 ± 0.2 days at 22°C); the invasive FAW also required less time to complete a generation (e.g., 20 days from egg to adult at 31°C compared with 23 days at 30°C); the number of eggs produced per mated female was also higher at all similar temperatures (1,432.7 ± 81.1 eggs at 31°C compared with 790.1 ± 105.3 eggs at 30°C; 1,671.7 ± 77.4 eggs at 25°C compared with 1,071.0 ± 80.6 eggs at 26°C; 1,555.0 ± 66.6 eggs compared with 993.5 ± 109.4 eggs at 22°C). Furthermore, the newly invasive FAW had higher larval and pupal survival rates (more than 96% and 86%, respectively, at 22, 25, 28, and 31°C) and a higher mating success rate (96%–100% at 22, 25, 28, and 31°C) (see Table 1). The excellent performance of the invasive FAW on corn leaves under constant temperature conditions suggests potentially severe future damage to this crop in China. Moreover, we observed the development time of 66 individuals of the newly invasive FAW that hatched on 29 July and experienced a mean daily temperature of 32.3°C and the fecundity of ten mated females under natural conditions by using the same rearing methods above. We found that the FAW exhibited excellent performance with 10.4 days larval period, 5.7 days for the pupal period, and 1,424 eggs per mated female. These results suggest that performance of life‐history traits tends to be better in the newly invasive FAW than the native FAW. Compared with the life‐history traits of the South Africa FAW (also fed on corn leaves) observed by Plessis et al. (2020, see their data in their Table 1), we found that the duration of pupal development of FAW was shorter in the current study at all similar temperatures (such as, 12.6 ± 0.1 days for females and 14.4 ± 0.1 days for males compared with 17.1 ± 0.24 days at 22°C).

More interestingly, we found that a small proportion of females delayed oviposition and began to lay eggs on the 7th to 9th day after adult emergence at all rearing temperatures. This suggests that these females do not mate with males in the first four days after emergence because mated females lay eggs either on the same day or the next night after mating. Thus, we speculate that these few females leave their location and migrate to elsewhere. It is known that migrants can be classified as either obligate or facultative (Dingle & Drake, 2007). The present study suggests that a fraction of the population may move away, while most of the population may remain in their breeding area. Of course, to confirm this hypothesis, we need to perform further experiments by using a tethered‐flight technique or to test ovarian development.

In the FAW, the pupal developmental time was significantly longer in males than females at all temperatures (Figure 1), resulting in the early emergence of females (protogyny). This protogyny phenomenon has been demonstrated in some lepidopteran species, such as the tobacco cutworm *Spodoptera litura* (Li et al., 2014), the cotton bollworm *Helicoverpa armigera* (Chen et al., 2014; Nunes et al., 2017), and the diamondback moth *Plutella xylostella* (Uematsu & Morikava, 1997). However, the biological reason for this emergence pattern is not well understood in insects. It may represent an evolutionary strategy to promote mating between individuals from distinct populations (Bento et al., 2006; Uematsu & Morikava, 1997) or it may be a strategy to reduce inbreeding (Li et al., 2014).

We found that 25°C conditions led to shorter larval development time but higher pupal weight than at 22 and 19°C (for larval development time: 16 days vs. 22 and 39 days; for female pupal weight: 163.9 mg vs. 154.2 mg and 159.4 mg; for male pupal weight: 181.3 mg vs. 173.3 mg and 177.9 mg, respectively) (see Table S1). Thus, the FAW did not follow the TSR. Furthermore, the fecundity was also highest at 25°C with a mean of 1,672 eggs per female. The shorter development times caused by higher temperatures result in larger body weights and higher egg production in the FAW, which clearly enhances its reproduction and results in serious damage to corn. Then, why do some insect species follow the TSR and some exhibit the reverse TSR? We speculate whether an insect species follows the TSR or not may be related to its diapause traits. Those species with summer diapause may exhibit the TSR, as indicated by the cabbage beetle, *Colaphellus bowringi* (Tang et al., 2017) and the cabbage butterfly, *Pieris melete* (Tang et al., 2019) because their reproductive periods occur in the spring and autumn and they have experienced strong selection for body size under relatively low environmental temperatures during the process of evolution. Those species with winter diapause triggered by shortening day lengths combined with high autumn temperatures may exhibit the reverse TSR, as indicated by the Asian corn borer, *O. furnacalis* (He et al., 2019; Xia et al., 2019; Xiao et al., 2016), and the rice stem borer, *Chilo suppressalis* (Fu et al., 2016; Huang et al., 2018). These two species enter winter diapause in response to high autumn temperatures and experienced strong selection for body size under warm conditions. The present study in the FAW suggests that migratory tropical insects may not follow the TSR, because the FAW experienced strong selection for body size under warm conditions. Similar reverse response was found in an invading population of the small cabbage white butterfly *Pieris rapae* (Kingsolver et al., 2007). However, to verify this speculation, additional insect species with similar characteristics should be investigated.

We found a significant positive relationship between pupal weight and larval development time at almost all temperatures, following the theory that animals with a longer growth period should be larger (Nijhout et al., 2010), that is, “one must grow for a longer time to get larger” (Roff, 2000). Thus, our results reveal that there is a trade‐off between the two traits. The relationship between development time and body size has been observed in 77% of Lepidopteran insect species (122 of 146 datasets) (Teder et al., 2014). However, a negative relationship between the two traits has been reported in the rice stem borer *C. suppressalis* reared under field conditions (Huang et al., 2018) and the Asian corn borer *O. furnacalis* reared at constant temperatures (Xia et al., 2019), in which a relatively shorter larval developmental time resulted in a relatively larger pupal weight.

The fecundity advantage hypothesis suggests that larger females produce more offspring than smaller females (Andersson, 1994; Darwin, 1874; Honek, 1993; Omkar & Afaq, 2013). Our data support this hypothesis. The FAW exhibited significant positive relationship between adult weight and adult fecundity at all temperatures (Figure 6). This significant positive relationship between pupal weight and adult fecundity has been reported in other lepidopteran species, such as the spruce budworm *Choristoneura fumiferana* (Miller, 1957), the sugarcane borer *Diatraea saccharalis* (Bessin & Reagan, 1990), the Mexican rice borer *Eoreuma loftini* (Spurgeon et al., 1995), the oriental armyworm *Mythmna separate* (Luo et al., 1995), the moth *Streblote panda* (Calvo & Molina, 2005), and the diamondback moth *Plutella xylostella* (Zhang et al., 2012).

Longevity is expected to involve a trade‐off with reproductive effort in most organisms (Bell & Koufopanou, 1986; Flatt, 2011; Harshman & Zera, 2007; Tatar, 2010); that is, reproduction tends to shorten the lifespan, the so‐called “cost of reproduction” (Williams, 1966). The present study in FAW supports this prediction; the females that reproduced more lived shorter lives (Figure 7). Such a negative relationship between the two traits has been recorded in the grasshoppers *Chorthippus brunneus* (de Souza Santos & Begon, 1987), the melon fly *Bactrocera cucurbitae* (Miyatake, 1997), the fruit fly *Drosophila melanogaster* (Flatt & Kawecki, 2007; Sambucetti et al., 2015), and the cotton bollworm *H. armigera* (Thyloor et al., 2016). However, little is known about the proximate mechanisms underlying these trade‐offs. Fecundity might reduce survival because of the costly production of gametes or survivorship might be decreased due to the elevated mortality risk associated with courtship and mating behavior (Bell & Koufopanou, 1986). In *Drosophila* and other insects, juvenile hormones have been proposed to stimulate reproduction at the expense of survival (Flatt et al., 2005; Tu et al., 2005).

The very high survival rates of larvae and pupae and the high mating success rates in the present experiments indicate that our rearing technique is very successful; that is, larvae reared individually in Petri dishes until adult eclosion and individual pairs of male and female moths placed in sealed plastic bags (20 × 30 cm) with cotton balls moistened in sugar solution for mating and oviposition. These rearing techniques may be adopted in other similar laboratory studies.

## 5 CONCLUSIONS

The newly invasive FAW showed excellent performance in its life‐history traits on corn leaves, with shorter development times, very high survival rates of larvae and pupae, high mating success rates, and fecundity. This performance shows that the FAW possesses traits that enable it to establish successfully in new environments and make it a new pest of corn production in China. Our study has also obtained some new information about the life‐history traits of the FAW, that is, a notable protogyny phenomenon; the TSR not being applicable; delayed preoviposition periods in a small proportion of females; a positive relationship between pupal weight and larval development time; a positive relationship between adult weight and fecundity and a negative relationship between fecundity and longevity. These findings can help us to predict the population dynamics of this new invasive pest on corn in the field and to develop a suitable practical management strategy.

## CONFLICT OF INTEREST

The authors declare that they have no conflicts interests.

## AUTHOR CONTRIBUTION

**Li‐Li Huang:** Investigation (equal); Methodology (equal); Writing‐original draft (lead). **Fangsen Xue:** Conceptualization (equal); Writing‐review & editing (lead). **Chao Chen:** Data curation (equal); Formal analysis (equal). **Xin Guo:** Investigation (equal). **Jianjun Tang:** Data curation (equal); Formal analysis (equal). **Ling Zhong:** Investigation (equal). **Hai‐Min He:** Conceptualization (equal); Investigation (equal); Methodology (equal).

## Supporting information

Figure S1

Figure S2

Table S1

## Data Availability

Empirical data have been archived in DataDryad: https://doi.org/10.5061/dryad.6djh9w111
