# Supplementary figures and images for: Effects of temperature on life‐history traits of the newly invasive fall armyworm, Spodoptera frugiperda in Southeast China
Source: Ecol Evol. 2021 Mar 18;11(10):5255–64. doi: 10.1002/ece3.7413 (PMC8131782; doi:10.1002/ece3.7413)

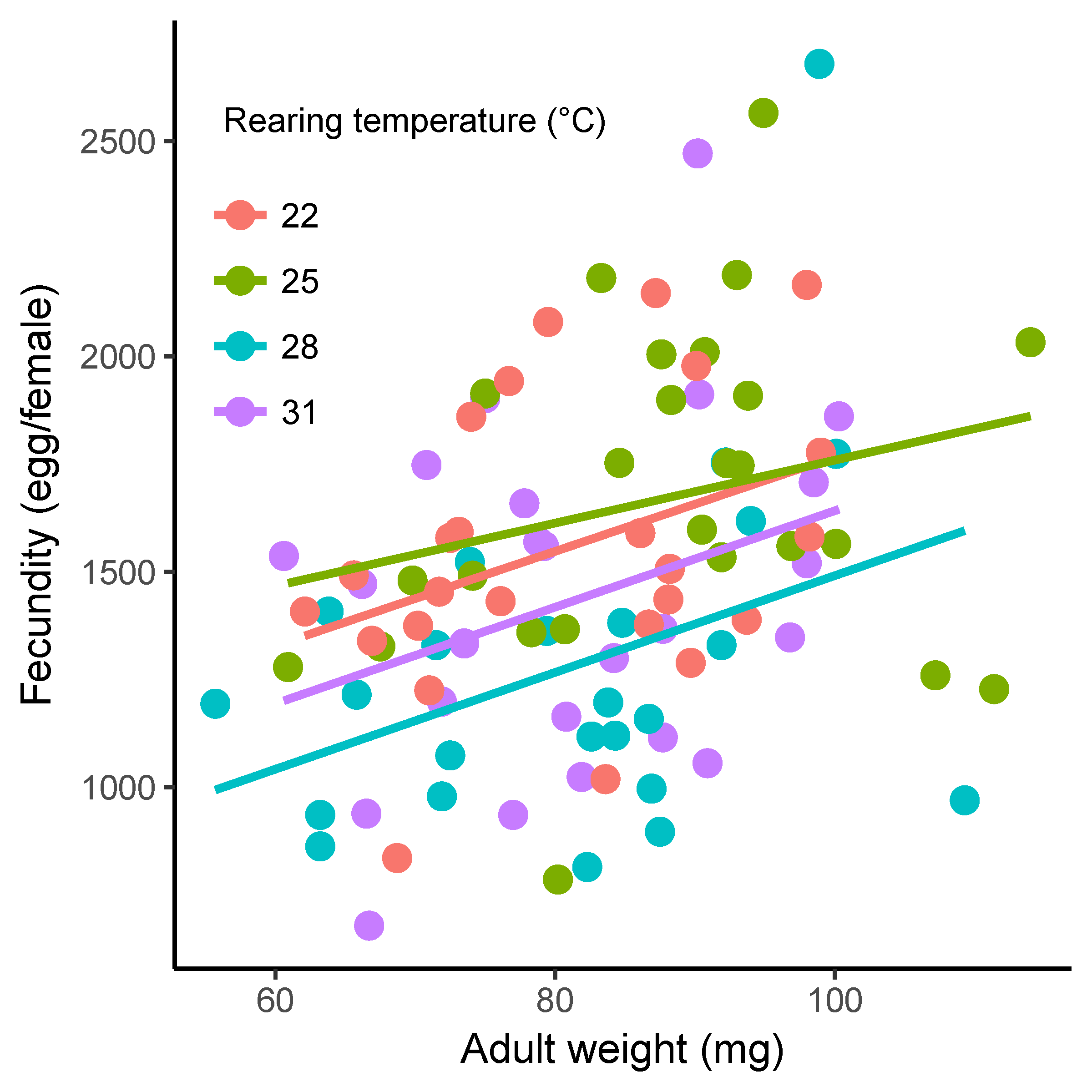

Supplement: Supplementary file 1 — Figure S1 [file ECE3-11-5255-s001.tif]

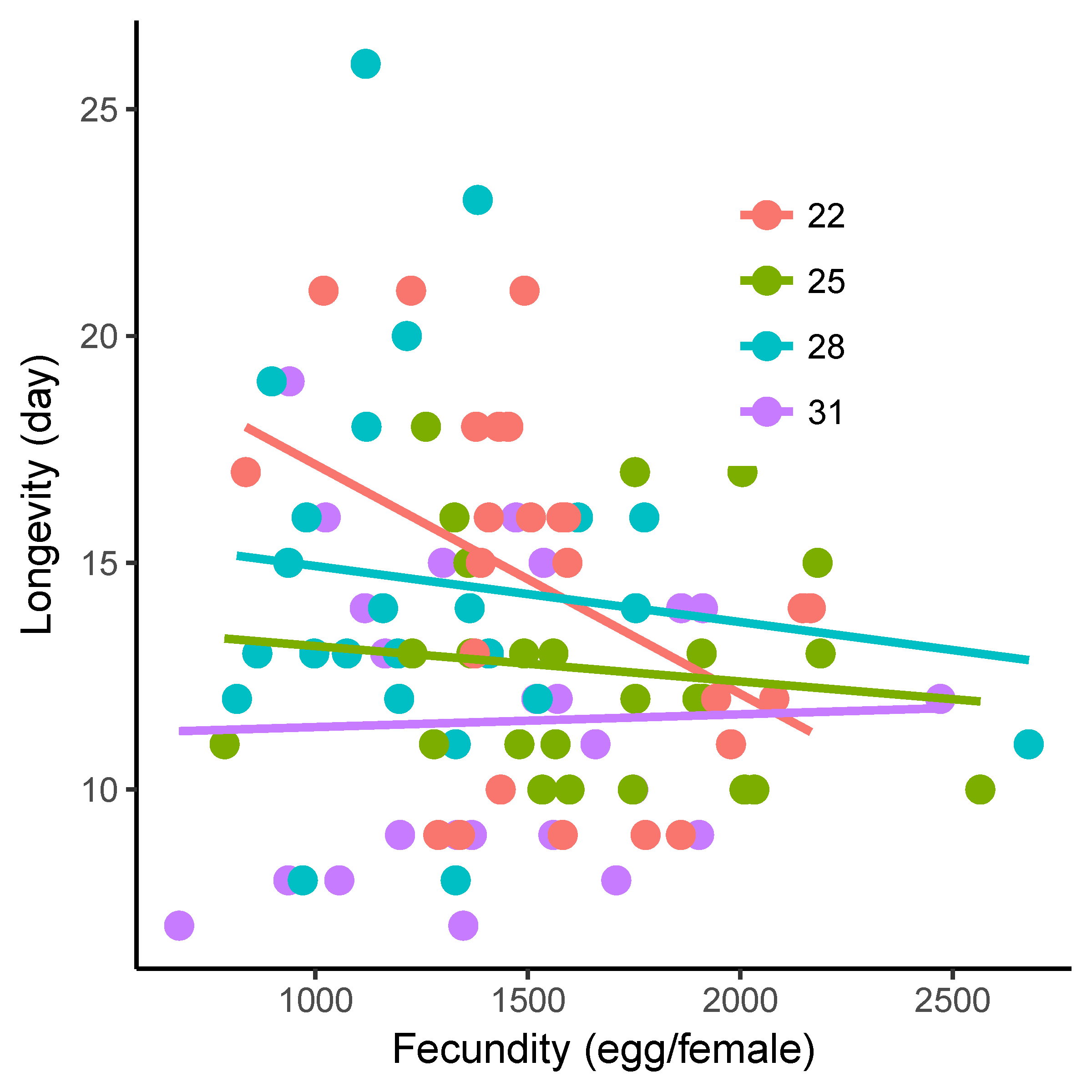

Supplement: Supplementary file 2 — Figure S2 [file ECE3-11-5255-s002.tif]
