# Supplementary material for: Effects of temperature on life‐history traits of the newly invasive fall armyworm, Spodoptera frugiperda in Southeast China
Source: Ecol Evol. 2021 Mar 18;11(10):5255–64. doi: 10.1002/ece3.7413 (PMC8131782; doi:10.1002/ece3.7413)
Supplement: Supplementary file 3 — Table S1 [file ECE3-11-5255-s003.doc]

**S1 Table** Life history data (mean ± SE.) for male and female *S. frugiperda* at different temperatures

| traits | 19 °C | |  | | 22 °C | |  | | 25 °C | |  | | 28 °C | |  | | 31 °C | |  |
| --- | --- | --- | --- | --- | --- | --- | --- | --- | --- | --- | --- | --- | --- | --- | --- | --- | --- | --- | --- |
|  | Female | Male | | Female | | Male | | Female | | Male | | Female | | Male | | Female | | Male | |
|  | N = 62 | N = 55 | | N = 132 | | N = 90 | | N = 137 | | N = 116 | | N = 118 | | N = 113 | | N = 111 | | N = 110 | |
| Larval time (d) | 38.6 ± 0.3a* | 39.9 ± 0.4a | | 21.5 ± 0.2b b | | 22.0 ± 0.2b | | 16.2 ± 0.1c | | 16.4 ± 0.1c | | 12.7 ± 0.1d | | 13.2 ± 0.1d | | 11.1 ± 0.1e | | 11.0 ± 0.1e | |
| Pupal time (d) | 21.1 ± 0.2 a* | 24.4 ± 0.2 a | | 12.6 ± 0.1 b* | | 14.4 ± 0.1 b | | 8.6 ± 0.0 c* | | 10.0 ± 0.1 c | | 6.8 ± 0.0 d* | | 7.3 ± 0.0 d | | 6.0 ± 0.0e* | | 6.9 ± 0.0 e | |
| Pupal weight (mg) | 159.4 ± 2.5 a* | 177.9 ± 2.6 a | | 154.2 ± 2.1 a* | | 173.3 ± 2.1 a | | 163.9 ± 0.01 a* | | 181.3 ± 1.6 a | | 159. 3 ± 1.9 a* | | 169.3±109b | | 157.8 ± 1.7 a | | 160.3 ±1.6 c | |
| Growth rate(In mg/d) | 4.1 ± 0.1 a | 4.5 ± 0.1 a | | 7.2 ± 0.1 b* | | 7.9 ± 0.1 b | | 10.2 ± 0.1 c* | | 11.1 ± 0.2 c | | 12.6 ± 0.1 d | | 12.9 ± 0.1 d | | 14.2 ± 0.1 e | | 14.6 ± 0.1 e | |
| Adult weight (mg) | 85.2±1.5 a* | 92.1±1.4 a | | 77.7 ± 0.9 a* | | 83.1 ± 1.0 a | | 81.6 ± 1.0 b* | | 86.6 ± 0.9 a | | 78.1 ± 1.2 a | | 76.6 ± 1.0 b | | 75.7 ± 1.0 a | | 74.4 ± 0.9 b | |
| weight loss (%) | 46.5 ± 0.5 a | 48.2 ± 0.4 a | | 49.5 ± 0.3 b* | | 51.9 ± 0.4 b | | 50.2 ± 0.3 bc* | | 52.2 ± 0.2 bc | | 51.1 ± 0.3 c* | | 54.7 ± 0.3 cd | | 52.1 ± 0.3 cd* | | 53.6 ± 0.4 d | |

Note: Values within one row followed by different letters are significantly different at 0.05 level based on one-way ANOVA and Tukey’s HSD multiple tests. * means

significantly different between sexes ( T test, *P* ﹤0.05)
